# Primary Thyroid Mucosa-Associated Lymphoid Tissue (MALT) Lymphoma: A Case Report

**DOI:** 10.7759/cureus.83842

**Published:** 2025-05-10

**Authors:** Nuno Gonçalves, Cristina Monteiro, Luísa Calais Pereira, Cláudia Lima, Inês Vaz Arnaud

**Affiliations:** 1 General Surgery, Unidade Local de Saúde do Alto Minho, Viana do Castelo, PRT

**Keywords:** autoimmune thyroiditis, case report, malt lymphoma, thyroid lymphoma, total thyroidectomy

## Abstract

Primary thyroid lymphoma (PTL) is a rare entity. Mucosa-associated lymphoid tissue (MALT) lymphoma represents only a small portion of all PTLs and is usually associated with chronic lymphocytic thyroiditis. Most cases are indolent and asymptomatic, which may delay diagnosis.

We present the case of a 63-year-old woman with a history of autoimmune hypothyroidism who developed a rapidly enlarging anterior neck mass and compressive symptoms. Ultrasound revealed an enlarged thyroid gland with a heterogeneous right lobe with a 34 mm lesion suggestive of focal thyroiditis. Fine-needle aspiration cytology was negative for malignancy (Bethesda II). Due to clinical progression, a total thyroidectomy was performed. Histopathological and immunohistochemical analyses confirmed MALT lymphoma.

Primary MALT lymphoma of the thyroid is a rare and often underrecognized diagnosis. It should be considered in patients with chronic thyroiditis and new or enlarging nodules. Surgical excision may be both diagnostic and therapeutic in localized disease. Postoperative surveillance remains essential due to the potential for systemic involvement.

## Introduction

Primary thyroid lymphoma (PTL) is a rare malignancy, representing 1% to 5% of all thyroid cancers [1]. It most commonly affects elderly women and is frequently associated with chronic autoimmune thyroiditis, particularly Hashimoto’s thyroiditis [2,3]. The most prevalent histological subtype is diffuse large B-cell lymphoma, accounting for 60% to 80% of PTL cases [4]. In contrast, extranodal marginal zone B-cell lymphoma of mucosa-associated lymphoid tissue (MALT) is far less common, comprising only 6% to 28% of cases [2].

MALT lymphoma of the thyroid typically presents as a painless, slowly enlarging neck mass, often in the setting of longstanding autoimmune thyroid disease [3-5]. Diagnosis may be challenging, as imaging and fine-needle aspiration (FNA) can be inconclusive [3]. Treatment strategies depend on the disease extent and include surgery, radiotherapy, or combined chemoradiotherapy [3,5-7].

We report a rare case of primary thyroid MALT lymphoma in a patient with autoimmune hypothyroidism, presenting with rapid gland enlargement and compressive symptoms, managed with total thyroidectomy.

## Case presentation

A 63-year-old woman with a six-year history of autoimmune hypothyroidism under levothyroxine replacement presented with a rapidly enlarging anterior neck mass and progressive compressive symptoms, including dysphagia and discomfort. She denied voice changes or weight loss.

Her past medical history included autoimmune hepatitis versus hepatic dysfunction secondary to hypothyroidism, sliding hiatal hernia, dyslipidemia, and a previous endoscopic variceal ligation.

Physical examination revealed an enlarged, firm, right thyroid lobe with poorly defined inferior borders. A smaller, elastic, and mobile contralateral nodule was also palpable.

**Figure 1:**
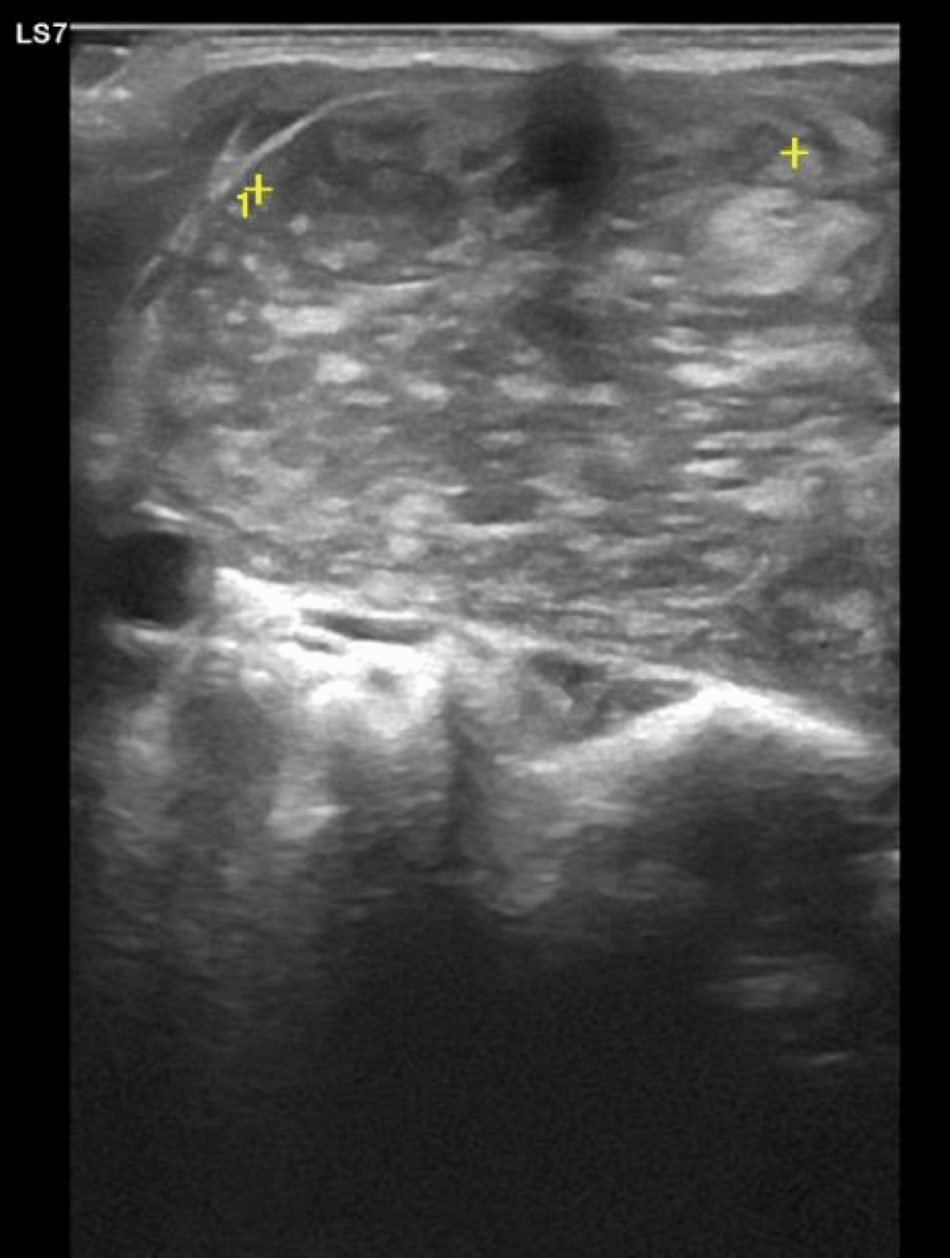
Thyroid ultrasound showing diffuse parenchymal heterogeneity and a vaguely nodular iso/hypoechoic area in the anterior portion of the right lobe, measuring approximately 34 mm.

Thyroid ultrasound demonstrated diffuse parenchymal heterogeneity and a vaguely nodular, iso to hypoechoic area in the anterior portion of the right lobe, measuring 34 mm, classified as EU-TIRADS 3. The left lobe showed a solid, echogenic nodule measuring 18 mm. No cervical lymphadenopathy was noted (Figure 1).

**Figure 2:**
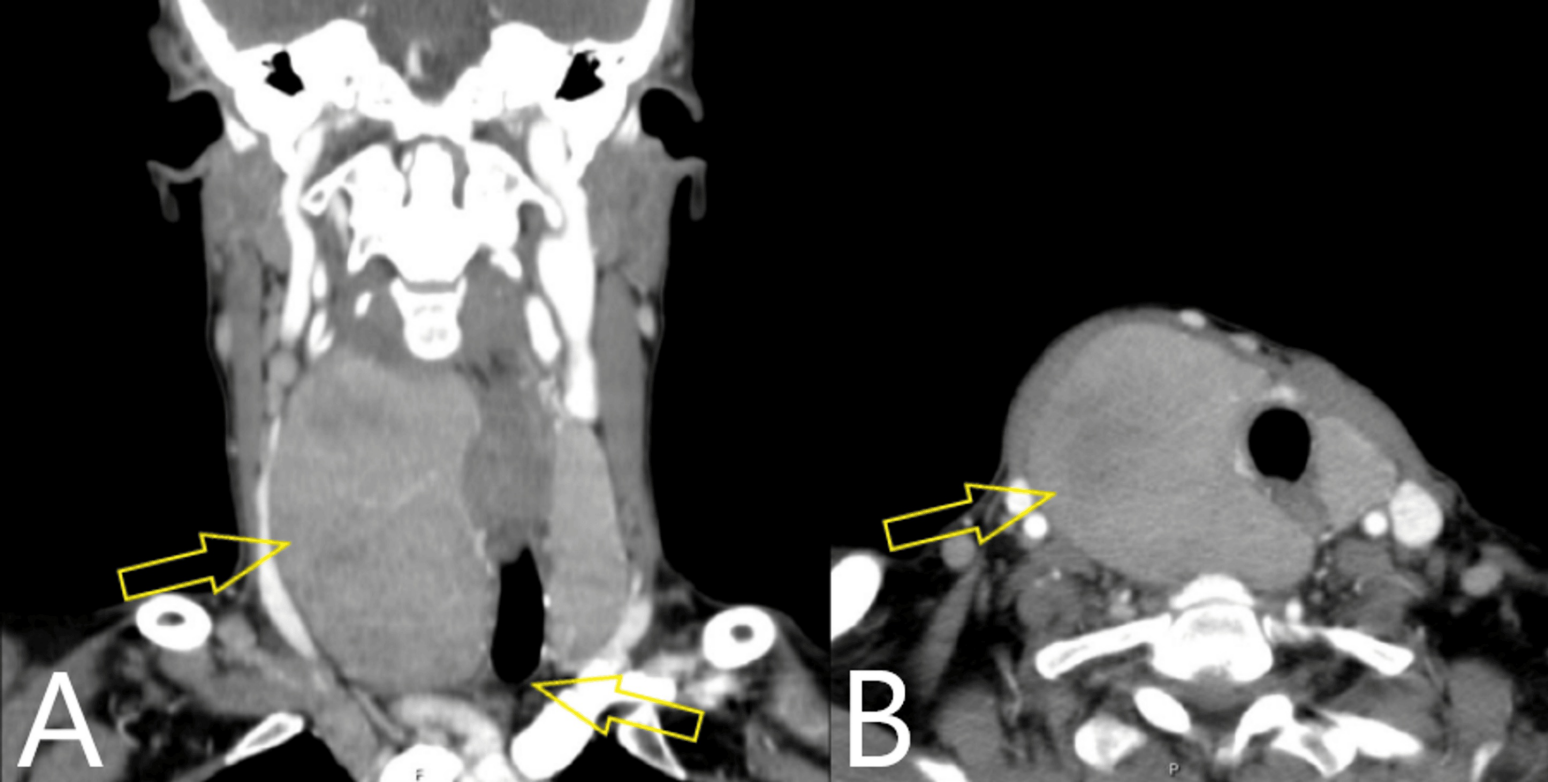
Coronal (A) and axial (B) CT images of the neck and chest showing an enlarged right thyroid lobe extending into the superior mediastinum with marked compression and deviation of adjacent structures including the trachea, esophagus, and cervical vessels (yellow arrows).

FNA cytology of the dominant lesion returned Bethesda II (benign). Due to the rapid clinical evolution and presence of compressive symptoms, total thyroidectomy was proposed. Preoperative contrast-enhanced computed tomography (CT) of the neck and chest revealed an enlarged right thyroid lobe with inferior extension into the superior mediastinum and deviation of adjacent structures, including the trachea and cervical vessels (Figure 2).

**Figure 3:**
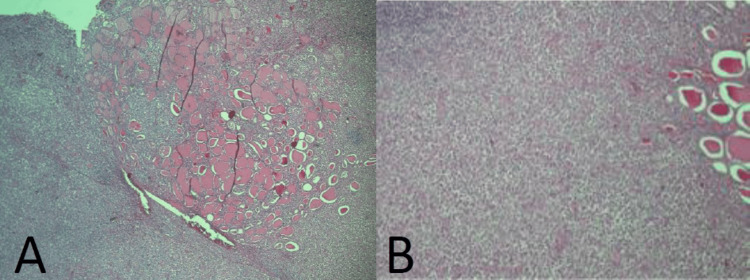
Histopathological examination of total thyroidectomy specimen: (A) (4x) Thyroid parenchyma surrounded by atypical lymphoid proliferation. (B) (10x) Atypical lymphoplasmacytic infiltrate involving thyroid follicles.

Histopathological analysis revealed a diffuse lymphoid proliferation with predominant plasmacytoid cells infiltrating thyroid follicles. Immunohistochemistry was positive for CD20, CD38, and MUM1 and negative for CD3, CD10, BCL6, and AE1/AE3. These findings were consistent with extranodal marginal zone B-cell lymphoma (MALT type) of the thyroid (Figure 3).

Postoperative recovery was uneventful. After directed hematological evaluation excluded systemic involvement, the patient was placed under multidisciplinary follow-up with surveillance. At one-year follow-up, she remained asymptomatic with no evidence of recurrence.

## Discussion

Primary thyroid MALT lymphoma is a rare subtype of extranodal marginal zone B-cell lymphoma and accounts for a small fraction of thyroid malignancies [2]. It is strongly associated with chronic lymphocytic thyroiditis, and its development is thought to result from chronic antigenic stimulation within lymphoid tissue ectopically present in the thyroid gland [3]. Most affected patients are older women with a long-standing history of Hashimoto’s thyroiditis [2].

Unlike diffuse large B-cell lymphoma, which often presents aggressively, MALT lymphomas tend to follow a more indolent course [3-5]. However, clinical presentation can vary, and some patients develop rapidly growing thyroid masses with compressive symptoms, as seen in our case. These symptoms may raise suspicion for anaplastic carcinoma or other aggressive neoplasms.

Diagnosis can be challenging. Ultrasound findings are often nonspecific, and FNA cytology may yield inconclusive or falsely benign results [3]. In this case, despite a Bethesda II result, clinical progression prompted surgical intervention, which ultimately led to the correct diagnosis.

Histopathological confirmation and immunophenotyping are essential for accurate diagnosis [3]. MALT lymphomas typically express CD20, CD79a, and other B-cell markers while lacking T-cell markers and high-grade lymphoma features [4].

There is no consensus on the optimal treatment for primary thyroid MALT lymphoma due to its rarity [3,5-7]. Options include surgery, radiotherapy, and systemic therapy depending on the disease stage. For localized disease, thyroidectomy or radiotherapy alone may be curative. In this case, total thyroidectomy was both diagnostic and therapeutic. As systemic disease was excluded, no adjuvant therapy was required.

This case reinforces the importance of maintaining a high index of suspicion for thyroid lymphoma in patients with autoimmune thyroiditis and rapidly enlarging nodules, even when cytology is benign. Surgical resection may be warranted in the presence of compressive symptoms or diagnostic uncertainty.

## Conclusions

Primary thyroid MALT lymphoma is a rare and often underdiagnosed condition, particularly in patients with chronic autoimmune thyroiditis. Its clinical and radiological features may mimic benign or other malignant thyroid disorders, and cytology alone may be insufficient for diagnosis. Surgical intervention can play a crucial role when compressive symptoms are present or when diagnostic uncertainty persists. Early recognition and appropriate management are essential to ensure favorable outcomes in this indolent but potentially challenging disease.
